# Importance of postmortem anthropometric evaluation in defining the role of malnutrition as a cause of infant and child deaths in Sub-Saharan Africa and South Asia: a cohort study

**DOI:** 10.1136/bmjopen-2024-089874

**Published:** 2025-02-17

**Authors:** Priya Mehta-Gupta Das, Zachary J Madewell, Dianna M Blau, Cynthia G Whitney, Usha Ramakrishnan, Aryeh D Stein, Melissa F Young, Parminder S Suchdev, Child Health and Mortality Prevention Surveillance Network

**Affiliations:** 1 Rollins School of Public Health, Emory University, Atlanta, Georgia, USA; 2 Global Health Center, Centers for Disease Control and Prevention, Atlanta, Georgia, USA; 3 Emory Global Health Institute, Emory University, Atlanta, Georgia, USA; 4 Hubert Department of Global Health, Emory University Rollins School of Public Health, Atlanta, Georgia, USA

**Keywords:** Epidemiology, Nutrition, Mortality, Community child health

## Abstract

**ABSTRACT:**

**Objectives:**

To evaluate how postmortem anthropometric malnutrition (PAM) measures align with expert panel attribution of malnutrition as a causal or significant condition in under-5 mortality (U5M).

**Design:**

Cohort study using data from the Child Health and Mortality Prevention Surveillance network, incorporating clinical records, postmortem anthropometrics, minimally invasive tissue sampling, clinical abstraction and verbal autopsy to determine multiple causes of death.

**Setting/participants:**

1405 deaths of children aged 1–59 months from six African countries between 2016 and 2023.

**Primary and secondary outcome measures:**

PAM was determined using z-scores from the WHO Child Growth Standards: underweight (weight-for-age<(−2)), wasting (arm circumference-for-age or weight-for-length<(−2)) and stunting (length-for-age <(−2)). Performance metrics (sensitivity (SE), specificity (SP) and positive predictive values (PPV)) were calculated to determine the alignment between PAM and expert panel attribution of malnutrition as a causal or significant condition to death.

**Results:**

Nearly 75% of cases demonstrated moderate-to-severe malnutrition by PAM, while expert panels attributed malnutrition in 41% of cases. Performance metrics varied across anthropometric indices: underweight exhibited the highest SE (89.7%), while wasting based on arm circumference had the highest SP (81.9%) and PPV (76.8%). Discrepancies between PAM classification and expert panel attribution differed significantly by site, age, location of death and preventability of death (p<0.05). Adjusted multivariate regression showed that expert panel attribution was more likely with increasing severity of PAM.

**Conclusions:**

The proportion of U5M attributable to malnutrition ranged between 41% (expert panel attribution) and 74% (PAM). Variability in classification underscores the need for monitoring and quality improvement measures to address discrepancies. Improved alignment between PAM and panel assessments is essential for accurately identifying malnutrition-related deaths and designing effective interventions to reduce U5M.

**STRENGTHS AND LIMITATIONS OF THIS STUDY**

- By integrating clinical records, verbal autopsies, minimally invasive tissue sampling and expert panel reviews, this study provides a thorough and systematic approach to identifying malnutrition’s role in under-5 mortality, capturing multiple causes of death.
- The study included a large and diverse sample with data from 1405 deaths across six high-mortality countries.
- Reliance on postmortem measurements without accompanying antemortem data restricts the ability to accurately assess premortem nutritional status and differentiate between malnutrition and terminal weight loss caused by illness, potentially leading to misclassification.
- Differences in the interpretation and application of malnutrition guidelines by expert panels across sites introduce inconsistency, challenging the comparability of malnutrition attribution in cause-of-death assessments.

## Introduction

 Over the past three decades, global progress has been made in reducing under-5 mortality (U5M), with total deaths dropping from 12.6 million in 1990 to 4.9 million in 2022.^1^ Despite this progress, significant regional disparities persist, with more than 80% of U5M occurring in South Asia and Sub-Saharan Africa.^2^,^4^ Additionally, costs associated with U5M are substantial, with an estimated gross domestic product loss in human capital exceeding $150 billion.^5^ The United Nations Sustainable Development Goal 3.2 calls for the elimination of preventable U5M and stillbirths by 2030; however, based on current trajectories, it is unlikely this goal will be met.^2^ Strategies that will ensure further reduction in U5M must be driven by timely, comprehensive data that captures where, why and how children die before their fifth birthday.

The WHO estimates that malnutrition, inclusive of anthropometric deficits (ie, underweight, wasting or stunting) and micronutrient malnutrition, contributes to nearly half of U5M.^6^ However, these estimations relied heavily on modelled data, systematic reviews, vital registration systems, verbal autopsies, medical certifications and multisite case-control studies.^7^,^9^ These data often assume a single underlying cause of death (CoD), may be subject to recall bias, and lack sensitivity (SE) and specificity (SP), thereby limiting our understanding of the contribution of malnutrition to U5M.

Furthermore, gaps remain in estimating the contribution of malnutrition to U5M due to imprecise or restrictive definitions of postmortem malnutrition and a lack of guidance on how to systematically assign malnutrition as a CoD on the death certificate.^23 9^,^14^ To address the need for systematic integration of malnutrition, a recent study published by the Minimally Invasive Tissue Sampling (MITS) Alliance CoD Working Group developed a framework for integrating malnutrition, assessed by anthropometry, in CoD coding among children under 5 years of age.^10^ Briefly, the guidelines suggest that malnutrition should be listed as a causal factor in the death if the decedent is severely wasted, while moderate wasting and any stunting should be listed as another significant condition in the death. Inconsistent attribution of postmortem anthropometric malnutrition (PAM) as either a causal or a contributing condition to death can lead to overestimation or underestimation of the contribution of malnutrition to U5M, which may have substantial policy and programmatic implications for the investment in and eradication of malnutrition globally. These guidelines were intended to provide a framework to support MITS and other mortality estimation systems with a systematic framework of assigning and interpreting malnutrition in death certification. To our knowledge, these guidelines have not been evaluated in detail.

Our study seeks to address the gaps in estimating the contribution of malnutrition to U5M by using data from the Child Health and Mortality Prevention Surveillance (CHAMPS) network. CHAMPS was established to understand the causes of mortality among children under 5 years of age in Sub-Saharan Africa and South Asia. Once parents and caregivers of the decedent provide consent to participate in CHAMPS, the study teams collect comprehensive data from multiple sources, including clinical abstraction, verbal autopsy, postmortem anthropometric measures and tissue sampling using MITS. Expert panels at each site, known as Determination of CoD (DeCoDe) panels, adjudicate the findings for each death to assign immediate, underlying, co-morbid conditions (within the causal chain) and other significant conditions associated with the chain of events (but outside of the causal chain) that resulted in the death of the child.

A recent CHAMPS study found that the proportion of U5M attributable to malnutrition was higher when examining PAM indices than when compared with expert panel attribution.^15^ These differences in the proportion of U5M attributable to malnutrition and the gaps outlined above motivated our study. In this study, we seek to examine what proportion of malnourished children based on PAM indices were attributed as malnourished by DeCoDe. We further sought to identify whether this proportion varied by type and severity of PAM as well as other key sociodemographic factors.

## Materials and methods

### Overview of data source

Details on the CHAMPS approach, sample selection, eligibility criteria, site characteristics and specimen and data collection methods have been published in detail elsewhere.^11^,^1416^ In brief, CHAMPS collects standardised, population-based surveillance data from sites in Bangladesh, Ethiopia, Kenya, Mali, Mozambique, Sierra Leone and South Africa. These sites were originally selected based on high child mortality with the purpose of elucidating and tracking preventable causes of U5M. MITS is conducted, and tissue samples are examined using microbiology and pathology techniques. CHAMPS teams also reviewed clinical records and interviewed parents using a standard verbal autopsy form. All available data are then reviewed by a DeCoDe panel, which strives to identify multiple CoDs and decrease subjectivity through a standardised approach of applying clinical and laboratory findings to diagnose the conditions contributing to death based on the completeness and SP of data.^11^ For DeCoDe, the panel determined whether the death was preventable based on available information, including demographic, clinical, pathological and microbiological data, WHO verbal autopsy,^22^ photography and anthropometric measurements. When the panel determined a death to be preventable, it referred specifically to the conditions immediately surrounding the child’s death, rather than the broader social context.^23^

### Inclusion criteria

For this analysis, our sample included infant and child deaths aged between 1 month and 59 months that had undergone both MITS and DeCoDe procedures (n=1415). All sites except Bangladesh were included in these analyses. The Bangladesh site primarily enrolled stillbirths and newborns, resulting in a small sample of children aged 1–59 months (n=10), and was, therefore, excluded from this analysis. Our final analytic sample size was n=1405 infant and child deaths.

### Postmortem anthropometric malnutrition

Site MITS technicians were trained in manual anthropometry to collect measures of recumbent length, weight and mid-upper arm circumference.^24^ Postmortem anthropometry has previously been found to be both feasible and reliable even in the presence of rigour mortis following routine training in the use of standard equipment.^24^ Wooden-length boards (ShorrBoard, Weigh and Measure, LLC, Maryland, USA), digital scales (Rice Lake Weighing Systems, Rice Lake, WI, USA) and standard tape measures (Weigh and Measure LLC, Maryland, USA) were used to ensure accurate measurement of recumbent length, weight and arm circumference, respectively.

### Exposure: type and severity of PAM

Our exposure of interest was the type and severity of PAM, including underweight, wasting and stunting. Underweight was based on weight-for-age z-scores (ZWEI). Wasting was evaluated using two different indices: mid-upper arm circumference-for-age z-scores (ZAC) and weight-for-length z-scores (ZWFL). Stunting was based on length-for-age z-scores (ZLEN). Z-scores were produced using the WHO Child Growth Standards.^25 26^

Based on our knowledge of the severity of illness and malnutrition in the study settings,^27^ we expanded our definition of the lower bound of biologically plausible values to include cases with z-scores, −10 SD. Severity of PAM across z-scores was defined using the following SD cutoffs: (−2≤SD<−1), (−3≤SD<−2) and (−10≤SD<−3). These cutoffs may be interpreted as “at risk”, “moderate” and “severe”, respectively. The reference category of none was defined using the WHO growth standards defined upper bound limits (−1 SD≤ZLEN<6 SD, −1 SD≤ZWFL, ZWEI or ZAC<5 SD).^25^ We also examined any malnutrition, which was a composite indicator of the four anthropometric indices, defined as −10≤ZLEN, ZWEI, ZWFL or ZAC<−2.

### Outcome: DeCoDe panel attributing malnutrition as a causal or other significant condition to death

Determination of the chain of events leading to death is based on the WHO’s International Classification of Diseases, 10th Revision, and the WHO application of International Classification of Diseases, 10th Revision (ICD-10), deaths during the perinatal period.^28 29^ Data collection procedures were approved by ethics committees in each CHAMPS site as well as Emory University (Emory IRB#: 91706).

CHAMPS ethical protocols are described in greater detail on the study website (https://champshealth.org/protocols/). Our outcome of interest was DeCoDe-attributed malnutrition as a causal or other significant condition contributing to death. The conditions and associated ICD-10 codes used for this definition included Kwashiorkor (ICD-10 Code=E40), nutritional marasmus (ICD-10 Code=E41), marasmic kwashiorkor (ICD-10 Code=E42), unspecified severe protein-calorie malnutrition (ICD-10 Code=E43), protein-calorie malnutrition of moderate and mild degree (ICD-10 Code=E44), moderate protein-energy malnutrition (ICD-10 Code=E44.0), mild protein-calorie malnutrition (ICD-10 Code=E44.1), retarded development following protein-calorie malnutrition (ICD-10 Code=E45), unspecified protein-calorie malnutrition (ICD-10 Code=E46) and HIV-related wasting syndrome (ICD-10 Code=B22.2).

### SE analyses

Since the mechanisms by which preterm birth (PTB), low birth weight (LBW) or small for gestational age (SGA) infants may die or are assigned as a CoD may differ from infants or older children lacking these conditions, we performed SE analyses to examine differences in sample demographics, modelling patterns and model fit statistics between analytic samples including and excluding possible PTB, LBW or SGA cases. We used data on gestational age (GA) and birthweight (BW) that were obtained by maternal or child clinical chart abstraction. PTB was defined using GA <37 weeks. We also considered an expanded definition of PTB that accounted for causes deemed to have “PTB complications” based on DeCoDe. LBW was defined as birth weight under 2500 g, and SGA was defined based on the sex-specific and GA-specific standards for growth determined by the International Fetal and Newborn Growth Consortium for the 21st Century (INTERGROWTH-21 study).^30^ SE analyses did not reveal any significant differences, therefore, we proceeded with the more inclusive sample of 1405 infant and child deaths to ensure sufficient power for statistical modelling.

### Data missingness

Data on BW (66.5% missing) and GA (99.0% missing), needed to classify cases as LBW, PTB or SGA, were missing for most of our analytic sample. When data onGA were available (n=14 cases), PTB based on GA alone was noted among all 14 cases. When we expanded the definition for PTB to include CoD information, we were able to classify an additional 107 deaths (total 121) as preterm. Of the 470 cases with birth weight data, we were able to classify 25.7% of the 470 cases as LBW. We were unable to classify any cases as SGA due to data missingness. We conducted SE analyses to evaluate the impact of missing data by comparing sociodemographic characteristics and malnutrition prevalence between samples with and without complete data on key variables (eg, sex, length/height and weight). No significant differences were observed, supporting our use of the most inclusive sample.

### Statistical analyses

Frequency distributions were used to describe demographic characteristics of our analytic sample. Pearson χ^2^ tests were used to examine relationships between covariates, exposure and outcomes of interest.

We used 2×2 contingency tables to evaluate the relationship between the presence or absence of PAM and DeCoDe panel-attributed malnutrition as a causal or another significant condition (Yes/No). For these analyses, the cutoff for the presence of PAM was defined using the following SD cutoffs (−10≤SD<−2), whereas no PAM was defined as ≥−2 SD and below the WHO-defined, index-specific upper bound.^25^ More specifically, we examined SE, SP and PPVs (which we also refer to as positive concordance) between the two methods. For the purposes of this analysis, our *a priori assumption* is that the expert panel (DeCoDe’s) attribution of malnutrition is the gold standard approach, as this method is based on multiple data points, including not only postmortem anthropometrics but also clinical records review and verbal autopsy summaries, which can elucidate whether malnutrition was present prior to onset of the fatal illness.

Covariates evaluated as potential confounders included age (in months), site and sex (male or female). We also examined the location of death (facility or community) as a potential confounder; however, the location of death was only associated with our outcome of interest and not other factors. Therefore, the final models were adjusted for site, age in months and sex.

Ordinal logistic regression was used to quantify the relationship between type (underweight, wasting based on ZAC, wasting based on ZWFL and stunting), the 3-level severity of PAM (at-risk, moderate and severe) and malnutrition, reported as a causal or other significant condition to the death of the child, as noted by DeCoDe panels. We used a p value<0.05 when exploring potential confounders and their associations with both exposure and outcome. Confounders were also considered if sufficient evidence in existing literature warranted inclusion in the model. Potential interactions across key variables were assessed to explore the relationship between PAM and the DeCoDe panel’s attribution of malnutrition, but inconsistencies in interaction patterns, including variability by site, age and sex, precluded justification for a stratified analysis. Model significance was evaluated using p<0.05. OR and 95% CI were reported. All analyses were completed in R statistical software (V.4.2.1, The R Foundation for Statistical Computing).

## Results

### Demographic characteristics of analytic sample

Between December 2016 and December 2023, 1405 infant and child deaths between 1 and 59 months of age underwent both MITS and DeCoDe, with complete data on sex, postmortem length and weight. Nearly a quarter of cases were from Sierra Leone (24.6%), with the remaining 23.8%, 22.0%, 17.9%, 6.5% and 5.3% from Kenya, South Africa, Mozambique, Mali and Ethiopia, respectively. There was a relatively even distribution by age in months and sex. Most deaths (71.8%) occurred in facilities (table 1).

**Table 1:** Demographics, classification of postmortem anthropometric measurements and attrition of deaths due to malnutrition for infant and child deaths enrolled in CHAMPS, from December 2016 to September 2023

	Analytic sample*, n=1405n (%)
Site	
Ethiopia	74 (5.3)
Kenya	334 (23.8)
Mali	91 (6.5)
Mozambique	251 (17.9)
Sierra Leone	346 (24.6)
South Africa	309 (22.0)
Age in months	
1–5 months	438 (31.2)
6–11 months	290 (20.6)
12–23 months	341 (24.3)
24–59 months	336 (23.9)
Female sex	631 (44.9)
Location of death†	
Community	394 (28.2)
Facility	1004 (71.8)
ZWEI‡	
Median (IQR)	−2.6 (3.3)
Severity of underweight‡	
None	331 (23.7)
At-risk (−2≤ZWEI‡<−1)	227 (16.3)
Moderate (−3≤ZWEI‡<−2)	214 (15.3)
Severe (−10≤ZWEI‡<−3)	623 (44.7)
ZAC§	
Median (IQR)	−1.6 (3.3)
Severity wasting based on ZAC§	
None	426 (36.8)
At-risk (−2≤ZAC§<−1)	224 (19.3)
Moderate (−3≤ZAC§<−2)	145 (12.5)
Severe (−10≤ZAC§<−3)	364 (31.4)
ZWFL¶	
Median (IQR)	−2.3 (3.2)
Severity of wasting¶	
None	355 (27.1)
At-risk (−2≤ZWFL¶<−1)	235 (17.9)
Moderate (−3≤ZWFL¶<−2)	213 (16.2)
Severe (−10≤ZWFL¶<−3)	508 (38.7)
ZLEN**	
Median (IQR)	−1.6 (3.1)
Severity of stunting**	
None	544 (39.7)
At-risk (−2≤ZLEN**<−1)	245 (17.9)
Moderate (−3≤ZLEN**<−2)	201 (14.7)
Severe (−10≤ZLEN**<−3)	379 (27.7)
Any malnutrition based on postmortem anthropometric z-scores	
At-risk to severe (−10≤ZWEI‡, ZAC§, ZWFL¶ or ZLEN**<−1)	1242 (88.4)
Moderate to severe (−10≤ZWEI‡, ZAC§, ZWFL¶ or ZLEN**<−2)	1045 (74.4)
Severe (−10≤ZWEI‡, ZAC§, ZWFL¶ or ZLEN**<−3)	794 (56.5)
DeCoDe-attributed malnutrition††	
Malnutrition recorded in causal chain or other significant condition, n (%)	572 (40.7)
Malnutrition recorded in the causal chain, n (%)	447 (31.8)
Malnutrition recorded as other significant condition, n (%)	127 (9.0)
Preventable death‡‡	
Yes, or possible under certain circumstances	1183 (85.5)

* Analytic sample defined as the number (n) of infants and children aged 1–59 months with complete data on length and weight at death;, includes those with possible history of preterm birthPTB, small for gestational ageSGA, or low birthweightLBW.

† Covariates have missing data: Llocation of death (n=7).

‡ Weight-for-age z-score (ZWEI) and severity of underweight missing (n=10), flagged as biologically implausible, those below – −10 or above upper limit of 5 for ZWEI.

§ Mid-upper arm circumference-for-age z-score (ZAC) and severity of wasting missing (n=246) due to WHO growth standards not able to calculate the z-score for those below 3 months^26 31^ or flagged as biologically implausible values, those below – −10 or above upper limit of 5 for ZAC.

¶ Weight-for-length z-score (ZWFL) and severity of wasting missing (n=94) due to WHO Ggrowth Sstandards not able to calculate the z-score for those with lengths below 45 cm 26 31 or those flagged as biologically implausible values, those below – −10 or above upper limit of 5 for ZWFL.

** Length -for-age z-score (ZLEN and severity of stunting missing (n=36), flagged as biologically implausible, those below – −10 or above upper limit of 6 for ZLEN;.

†† Determination of Cause of Death (DeCoDe) expert panels analyzeanalyse all available individual information, including laboratory, histopathology, abstracted clinical records, and verbal autopsy findings for each death. The DeCoDe site panel ascertains the underlying cause (event that precipitated the fatal sequence of events) and other antecedent, immediate, and maternal causes of deathCoD in accordance with the International Classification of Diseases, 10thTenth Revision, and the World Health OrganizationWHO death certificate. Two deaths had malnutrition recorded in the causal chain and as other significant condition; therefore, the sum is 574.

‡‡ Preventable death was redefined as a dichotomous variable: the categories of “yes” and “possible” were combined, and “non-consensus” and missing data were removed from the denominator (n=21).

BWbirthweightCHAMPSchild health and mortality prevention surveillanceCoDcause of deathDeCoDedetermination of cause of deathGAgestational ageLBWlow birth weightPTBpreterm birthSGAsmall for gestational ageZACmid-upper arm circumference-for-age z-scoresZLENlength-for-age z-scoresZWEIweight-for-age z-scoresZWFLweight-for-length z-scores

Overall, postmortem anthropometric data revealed that 1045 (74.4%%) deaths had moderate to severe PAM and 794 (56.5%) had severe PAM (table 1). A total of 60.0%, 43.9%, 54.9% and 42.4% were moderately to severely underweight, wasted based on ZAC, wasted based on ZWFL and stunted, respectively.

The DeCoDe panel attributed malnutrition as a cause or contributor to death for 40.7% of cases (table 1). Malnutrition was considered a causal factor in 447 (31.8%) deaths and other significant conditions in 127 (9.0%) deaths. Over 8 in 10 deaths (85.5%) were deemed preventable.^23^ Malnutrition was most likely to be recognised as an underlying cause (345/1405; 24.6%) when listed in the causal chain. Malnutrition was determined to be an immediate or morbid CoD in only 2 (0.1%) and 103 (7.3%) cases, respectively (online supplemental table 1).

### Contingency tables and evaluating performance metrics

Underweight correctly identified 89.7% (95% CI: 86.9, 92.1) of cases with DeCoDe-attributed malnutrition (table 2) but lacked SP (60.6; 95% CI: 57.2, 64.0) and PPV (61.3; 95% CI: 57.9, 64.6).

**Table 2:** Performance of various postmortem anthropometric indices to determine malnutrition as a causal or other significant condition among infant and child deaths in the CHAMPS network, December 2016 to September 2023

	DeCoDe-attributed malnutrition*				
	Yes	No	SE†	SP†	Positive concordance†
Underweight‡n=1395					
Yes	513	324	89.7 (86.9, 92.1)	60.6 (57.2, 64.0)	61.3 (57.9, 64.6)
No	59	499			
Wasted based on mid-upper arm circumference§n=1159					
Yes	391	118	77.0 (73.1, 80.6)	81.9 (78.7, 84.8)	76.8 (72.9, 80.4)
No	117	533			
Wasted based on weight-for-length¶n=1311					
Yes	453	268	82.7 (79.2, 85.7)	64.9 (61.4, 68.3)	62.8 (59.2, 66.4)
No	95	495			
Stunted**n=1369					
Yes	340	240	60.5 (56.3, 64.6)	70.3 (60.7, 73.4)	58.6 (54.5, 62.7)
No	222	567			
Any moderate to severe malnutrition††					
Yes	556	489	97.2 (95.5, 98.4)	41.3 (37.9, 44.7)	53.2 (50.1, 56.3)
No	16	344			

* Determination of Cause of Death (DeCoDe) expert panels analyzeanalyse all available individual information, including laboratory, histopathology, abstracted clinical records, and verbal autopsy findings for each death. Using this information, the site panel ascertains the underlying cause (event that precipitated the fatal sequence of events) and other antecedent, immediate, and maternal causes of death in accordance with the International Classification of Diseases, Tenth10th Revision, and the World Health OrganizationWHO death certificate.

† 2x2 contingency table comparing exposures and outcome of interest. SE: Sensitivity (%) = [(A)/(A+C)]*100, SP: Specificity (%) = [(D)/(B+D)]*100; Positive Concordance or positive predictive value (%) = [(A)/(A+B)]*100. certificate.^40^

**Table:** 

A (true positive)	B (false positive)	**PositiveConcordanceA/(A+B)**
C (false positive)	D (true negative)	
**Sensitivity A/(A+C)**	**Specificity D/(B+D)**	

[Bibr R40]

‡ Underweight is defined as a low weight-for-age z-score (ZWEI; Yes: -10 ≤ ZWEI< -2, No: -2 ≤ ZWEI ≤ 5); missing data (n=10), flagged as biologically implausible, those below – 10 or above upper limit of 5 for ZWEI.^26 31^

§ Wasted was evaluated based on a mid-upper arm circumference-for-age z-score (ZAC; Yes: -10 ≤ ZAC< -2, No: -2 ≤ ZAC ≤ 5); missing data (n=246), due to WHO Growth Standards not able to calculate z-score for those below 3 months^26 31^ or flagged as biologically implausible values, those below – 10 or above upper limit of 5 for ZAC.

¶ Wasted was also evaluated as a weight-for-length z-score (ZWFL; Yes: -10 ≤ ZWFL < -2, No: -2 ≤ ZWFL ≤ 5); missing data (n=94) due to WHO Growth Standards not able to calculate z-score for those with lengths below 45 centimeters^26 31^ or flagged as biologically implausible values, those below – 10 or above upper limit of 5 for ZWFL.

** Stunting is defined as length -for-age z-score (Yes: -10 ≤ ZLEN< -2, No: -2 ≤ ZLEN ≤ 6); missing data (n=36), flagged as biologically implausible, those below – 10 or above upper limit of 6 for ZLEN.

†† Any Moderate to Severe Malnutrition “Yes” was defined as (-10 ≤ any index < -2 SD), including: ZLEN or ZWEI or ZWFL or ZAC. The “No” category included those cases above or equal to -2 but less than the WHO-defined upper limit for the specific anthropometric index.^25^

CHAMPSChild Health and Mortality Prevention SurveillanceDeCoDedetermination of cause of deathPPVpositive predictive valueSEsensitivitySPspecificityZACmid-upper arm circumference-for-age z-scoresZLENlength-for-age z-scoresZWEIweight-for-age z-scoresZWFLweight-for-length z-scores

Wasting, based on ZAC, had a high SE (77.0%; 95% CI: 73.1, 80.6), SP (81.9%; 95% CI: 78.7, 84.8) and positive concordance (or PPV) (76.8%; 95% CI: 72.9, 80.4) between PAM and DeCoDe (table 2).

Wasting, based on ZWFL, had higher SE (82.7%; 95% CI: 79.2, 85.7) compared with wasting based on ZAC but at the expense of SP (64.9%; 95% CI: 61.4, 68.3). Positive concordance (or PPV) between PAM and DeCoDe was 62.8% (95% CI: 59.2, 66.4; table 2).

Overall performance metrics for stunting were the lowest relative to the other individual anthropometric indices. Stunting by PAM correctly identified 60.5% (95% CI: 56.3, 64.6) of deaths with DeCoDe-attributed malnutrition and correctly identified 70.3% (95% CI: 60.7, 73.4) of cases where DeCoDe did not attribute to malnutrition as a causal or significant condition to death. Nearly 59% (PPV: 58.6; 95% CI: 54.5, 62.7) of deaths with stunting also had DeCoDe-attributed malnutrition as a causal or significant condition to death (table 2).

The composite indicator of any moderate to severe PAM was highly sensitive (97.2%; 95% CI: 95.5, 98.4) but not specific (41.3%; 95% CI: 37.9, 44.7). Only about half of the cases with any PAM also had DeCoDe-attributed malnutrition (PPV: 53.2%; 95% CI: 50.1, 56.3).

### Factors associated with positive concordance between any postmortem malnutrition and DeCoDe attributing malnutrition as a causal or significant condition to death

Concordance of wasting by PAM or PAM by any measure and DeCoDe attribution of death differed by site, age in months, location of death and whether the death was preventable (table 3). There were no differences in concordance by sex. Further, the lead author (PMGD) and a paediatrician (PSS) took a closer examination of the clinical records of a random sample of 50 discordant cases who had evidence of PAM but not DeCoDe-attributed malnutrition to qualitatively evaluate whether malnutrition or poor growth were noted as potential contributors to the clinical course leading to death. Of these, 22% (11/50) had indications of malnutrition or poor feeding practices noted in the clinical record.

**Table 3:** Demographic characteristics of positive concordance* between moderate to severe wasting or any PAM and attribution of malnutrition as a causal or other significant condition by the DeCoDe panel, December 2016 to September 2023

Characteristic	Positive concordance* between wasting† and DeCoDe, n=**453**	P value‡	Positive concordance* between any PAM and DeCoDe, n=**556**	P value‡
Site, n (%)				
Ethiopia	43/44§ (97.7)	<0.001	64/68 (94.1)	<0.001
Kenya	141/203 (69.5)		153/248 (61.7)	
Mali	31/58 (53.4)		32/71 (45.1)	
Mozambique	67/104 (64.4)		99/186 (53.2)	
Sierra Leone	135/189 (71.4)		150/245 (61.2)	
South Africa	36/123 (29.3)		58/227 (25.6)	
Age in months, n (%)		<0.001		<0.001
1–5 months	93/223 (41.7)		124/364 (34.1)	
6–11 months	131/174 (75.3)		147/208 (70.7)	
12–23 months	145/192 (75.5)		172/251 (68.5)	
24–59 months	84/132 (63.6)		113/222 (50.9)	
Sex, n (%)		0.15		0.25
Female	209/318 (65.7)		256/464 (55.2)	
Male	244/403 (60.5)		300/58 (51.6)	
Location of death¶, n (%)		0.015		<0.001
Community	156/229 (68.1)		185/295 (62.7)	
Facility	297/489 (60.7)		371/746 (49.7)	
Preventable death¶ (Yes/No), n (%)		<0.001		<0.001
Yes/possible under certain circumstances	421/624 (67.5)		511/888 (57.5)	
No	30/87 (34.5)		42/143 (29.4)	

* Positive concordance was defined as DeCoDe attributing malnutrition as a cause or significant condition to death and wasting at death. Wasting was defined as aweight-for-length z-score (ZWFL; (Yes: −10≤ZWFL<−2, and No: −2≤ZWFL≤ 5); missing data (n=94) due to WHO Ggrowth Sstandards not able to calculate the z-score for those with lengths below 45 cm^26 31^ or flagged as biologically implausible values, those below – −10 or above the upper limit of 5 for ZWFL. Positive concordance was defined as DeCoDe attributing malnutrition as a cause or significant condition to death and the presence of any moderate to severe malnutrition “Yes” was defined as (−10≤any index < −2 SD), including: ZLEN, or ZWEI or, ZWFL or ZAC. The “No” category included those cases above or equal to −2 but less than the WHO-defined upper limit for the specific anthropometric index.^25^ Positive concordance or PPV (%)=((A)/(A+B))×100 (see table 2 footnote).^40^

† Wasting was defined as a weight-for-length z-score(ZWFL; (Yes: −10≤ZWFL<−2, No: −2≤ZWFL≤5); missing data (n=94) due to WHO Ggrowth Sstandards not able to calculate the z-score for those with lengths below 45 cm^26 31^ or flagged as biologically implausible values, those below – −10 or above upper limit of 5 for ZWFL.

‡ Associations between covariates were examined using Pearson’s Chi-squaredχ2 test (normally distribution), or Wilcoxon rank sum test (where the assumption of a normal distribution is violated) or Fisher’s exact test (2×2 contingency table or when samples sizes are small).

§ Demographic characteristic-specific 2×2 tables: (A/A+B)..Covariates have missing data: Location of death (); Preventable was redefined as a dichotomous variable: the categories of yes and possible were combined and non-consensus and missing data were removed from the denominator ().

¶ Covariates have missing data: location of death (n=4); preventable death was redefined as a dichotomous variable: the categories of “yes” and “possible” were combined, and “non-consensus” and missing data were removed from the denominator (n=14).

DeCoDedetermination of cause of deathPAMpostmortem anthropometric malnutritionPPVpositive predictive valueZACmid-upper arm circumference-for-age z-scoresZLENlength-for-age z-scoresZWEIweight-for-age z-scoresZWFLweight-for-length z-scores

### Ordinal logistic regression

**Figure 1:**
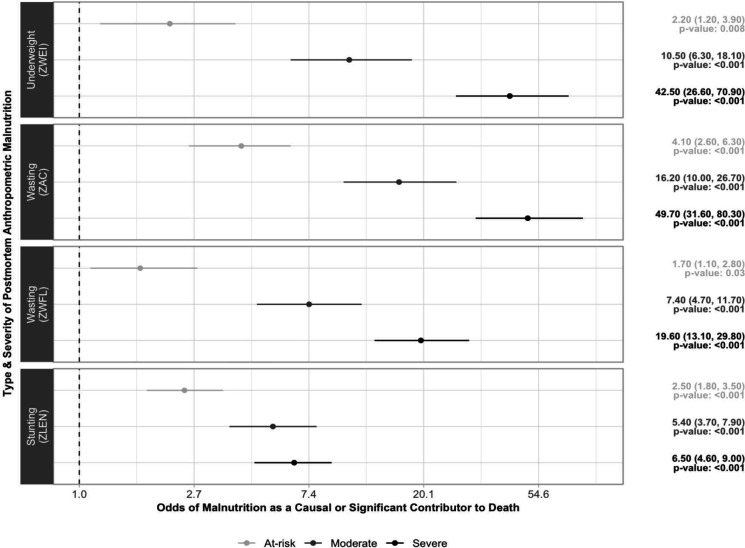
Adjusted odds of malnutrition* recognised by DeCoDe† panel as a causal or other significant condition† by type and severity of postmortem anthropometric malnutrition‡ at death. *The x-axis is shown on the log scale. Associations were examined using ordinal logistic regression models comparing the exposure (severity of various postmortem anthropometric indices) and outcome (expert panel attributing malnutrition as a cause or other significant conditions to death) adjusting site, sex and age. The reference group for the exposure varies by panel. Panel 1 examines the relationship between the severity of underweight based on ZWEI and malnutrition attributed by DeCoDe. ZWEI were classified as at-risk (−2≤ZWEI<−1), moderate (−3≤ZWEI<−2) or severe (−10≤ZWEI<−3). The reference group was not underweight (−2≤ZWEI≤5). Panel 2 examines the relationship between the severity of wasting based on ZAC and malnutrition attributed to DeCoDe. ZAC were classified as at-risk (−2≤ZAC<−1), moderate (−3≤ZAC<−2) or severe (−10≤ZAC<−3). The reference group was not wasted (−2≤ZAC≤5). Panel 3 examines the relationship between the severity of wasting based on ZWFL and malnutrition attributed to DeCoDe. ZWFLs were classified as at-risk (−2≤ZWFL<−1), moderate (−3≤ZWFL<−2) or severe (−10≤ZWFL<−3), with ZWFL to the reference group not wasted (−2≤ZWFL≤5). Panel 4 examines the relationship between the severity of stunting based on ZLEN and malnutrition attributed to DeCoDe. ZLEN were classified as at-risk (−2≤ZLEN<−1), moderate (−3≤ZLEN<−2) or severe (−10≤ZLEN<−3), with ZLEN to the reference group not stunted (−2≤ZLEN≤6). †DeCoDe expert panels analyse all available individual information, including laboratory, histopathology, abstracted clinical records and verbal autopsy findings for each death. Using this information, the site panel ascertains the underlying cause (event that precipitated the fatal sequence of events) and other antecedent, immediate and maternal causes of death in accordance with the International Classification of Diseases, 10th Revision, and the WHO death certificate. ‡Additional details on the type and severity of postmortem anthropometric indices are as follows: ZWEI and severity of underweight missing (n=10), flagged as biologically implausible, those below −10 or above the upper limit of 5 for ZWEI; ZAC and severity of wasting missing (n=246) due to WHO growth standards not able to calculate the z-score for those aged below 3 months^26 31^ or flagged as biologically implausible values, those below −10 or above the upper limit of 5 for ZAC; ZWFL and severity of wasting missing (n=94) due to WHO growth standards not able to calculate the z-score for those with lengths below 45 cm^26 31^ or flagged as biologically implausible values, those below −10 or above the upper limit of 5 for ZWFL; and ZLEN and severity of stunting missing (n=36), flagged as biologically implausible, those below −10 or above the upper limit of 6 for ZLEN. DeCoDe, determination of cause of death; ZAC, mid-upper arm circumference-for-age z-scores; ZLEN, length-for-age z-scores; and ZWEI, weight-for-age z-scores; ZWFL, weight-for-length z-scores.

Figure 1 illustrates the association between type (underweight, wasting based on ZAC, wasting based on ZWFL and stunting) and 3-level severity (at-risk, moderate, severe) of various PAM indicators and the odds of malnutrition being reported as a causal or significant condition in the death. In general, increasing severity of PAM was associated with greater odds of DeCoDe panel attributed malnutrition as a causal or significant condition to death.

There were no substantive differences between crude and adjusted models that controlled for age, site and sex. The severity of malnutrition based on ZWEI, ZAC, ZWFL and ZLEN was all independently associated with malnutrition as a causal or significant condition in death (figure 1). We observed a gradient association between exposure and outcome, indicating that greater severity of PAM was associated with higher adjusted odds of malnutrition being recognised by DeCoDe.

Underweight and wasting, based on ZAC, exhibited a similar stepwise pattern of increasing severity of PAM, being associated with increasing adjusted odds of DeCoDe attributing malnutrition as a causal or other significant condition in the death. These findings suggest that changes in weight and arm circumference may be most useful when assessing the adjusted odds of malnutrition being recognised by DeCoDe (figure 1).

Wasting, based on ZWFL, revealed a complementary gradient pattern; however, the magnitudes of effect were lower than wasting based on ZAC (figure 1). Stunting at death was also associated with significantly elevated odds of malnutrition being attributed as a causal or significant condition in the death, however, the magnitude of effects for moderate stunting and severe stunting were lower, relative to the other indicators of PAM. We also examined a multivariate model which included all measures of PAM. The inclusion of all metrics did not alter the pattern described in detail above.

## Discussion

This multisite study analysed over 1400 infant and child deaths from six high-mortality countries and found a high prevalence of malnutrition, as determined by postmortem anthropometry. Nearly three-quarters of the cases exhibited moderate to severe malnutrition, while more than half were characterised as severely malnourished. Despite PAM being common, DeCoDe panels attributed malnutrition as a CoD in only 4 in 10 deaths, resulting in an overall positive concordance between PAM and DeCoDe of 53%. This discordance could stem from weight loss during the terminal stages of illness or postmortem changes; however, the lack of antemortem measurements in most cases limits our ability to assess such changes. Given the short window between death and the MITS examination (within 72 hours postmortem), substantial postmortem changes in weight are unlikely.

Performance metrics varied by anthropometric indices; underweight exhibited the highest SE (89.7%), while wasting based on mid-upper arm circumference showed the highest SP (81.9%) and PPV (76.8%). We also noted differences in positive concordance between PAM and DeCoDe, especially wasting, which was specified in the MITS Alliance Guidelines^10^ to always be included in DeCoDe panel attribution of malnutrition as a causal or other significant condition by site. This variability may reveal differences in how DeCoDe panels may be interpreting anthropometry when classifying a CoD. These findings may further indicate the value of continued quality assurance and monitoring of consistent CoD attribution between site-specific DeCoDe panels.

Differences in positive concordance were also noted by age, with deaths in younger infants having lower positive concordance than those aged 6 months and above. These findings may reflect challenges with small measurements taken from younger infants who were born prematurely or SGA but not malnourished. Alternatively, these differences in concordance by age may be more indicative of maternal nutritional status, highlighting the need for comprehensive data collection on maternal nutritional status during pregnancy as future CHAMPS pregnancy surveillance studies are considered. Furthermore, these findings highlight the importance of incorporating data on gestational age and birth weight into malnutrition assessment. On further examination of information collected from clinical records for a random sample of 50 discordant cases, we found that 22% (11/50) had indications of malnutrition or poor feeding practices, suggesting that malnutrition may have been indirectly involved in that fraction. It is challenging to know why discordance existed between PAM and DeCoDe; the role of the DeCoDe panel is complicated and nuanced, taking many factors into their decisions. Future investigation into these discordant cases is needed to understand these decisions.

As one might expect, adjusted multivariate regression analysis revealed higher odds of expert panel attribution of death to malnutrition with increasing severity of PAM. This was particularly true for underweight and wasting as compared with stunting, suggesting that changes to arm circumference and weight may be most predictive of concordance between DeCoDe and PAM.

It is important to note that ZAC does not capture all cases in our analytic sample. ZAC can only be determined for children aged 3–59 months, while raw measurement cutoffs for arm circumference are applicable only to children aged 6–59 months.^26 31^ To classify the majority of our analytic sample, we chose to examine ZAC rather than the raw measurements of mid-upper arm circumference.

We found wasting based on ZAC to be most predictive of malnutrition-specific deaths, followed by underweight, and then wasting based on ZWFL. These findings support existing literature that highlights the importance of monitoring wasting through the measurement of arm circumference for mortality risk.^32^,^35^ Underweight revealed a similar gradient effect with larger magnitudes of effect when compared with wasting (based on ZWFL) and stunting. These results suggest that attribution of malnutrition by DeCoDe may be influenced by changes in ZAC and underweight. Biologically, these results highlight that as a child near death, they are likely to lose weight rather than experience declines in stature. Our findings among underweight cases complement other studies that found that monitoring underweight may be especially meaningful when identifying “small and nutritionally at-risk infants<6 months”^33 36 37^ perhaps most similar to the cases represented in CHAMPS. Frequent monitoring of weight and arm circumference may be critical when deciding when to intervene with evidence-based and potentially lifesaving nutrition interventions.

### Strengths and limitations

A key strength of this study was the ability to capture multiple causes of death, unlike previous studies that relied heavily on modelled or reviewed data from interventional or observational studies. Malnutrition was most likely to be recognised by DeCoDe panels as an underlying cause or other significant condition to death. It is, however, important to note that in some cases malnutrition was found to be an immediate cause or comorbid CoD. These findings would only be captured within a mortality surveillance system that captures multiple causes of death, thus highlighting one of the key benefits of the CHAMPS DeCoDe process.

In addition, causes of death were captured through the comprehensive, standardised data collection and DeCoDe process in association with anthropometric measurements taken using standardised anthropometric equipment and routinely trained personnel at the time of each MITS procedure. These measures enable more accurate and inclusive estimates of deaths attributed to malnutrition.^24^ While the inclusion of data from six high-mortality countries enhances generalisability to similar settings in Sub-Saharan Africa, the cohort may not fully represent all under-5 deaths within these regions or globally. Facility-based deaths are overrepresented due to CHAMPS’ reliance on timely death identification for MITS, potentially biasing findings towards families with better healthcare access. Additionally, variability in site-specific epidemiology and malnutrition attribution by DeCoDe panels may limit the comparability of findings across sites.^38^

Our study is also subject to limitations. First, because measurements are taken only at the time of death, the measurement data alone does not allow inference of causality. Second, we cannot control the variability of antemortem data, which would have been helpful for determining more clearly if malnutrition had been present before the fatal illness. We also lacked data on gestational age and birth weight for many cases, critical for distinguishing between LBW, PTB, SGA and intrauterine growth restriction. It is possible that we are measuring the relative validity of PAM against DeCoDe death attribution as the DeCoDe panels consider postmortem anthropometric measures in CoD attribution. CHAMPS teams have a limited window between death and burial to enrol and sample deaths, leading to potential challenges in enrolling deaths outside catchment facilities^11 21 39^; whether deaths not enrolled in CHAMPS are more or less likely to die from malnutrition than those who are enrolled needs further evaluation. Lastly, it is important to acknowledge the limitations of interpreting postmortem anthropometry in a cohort of severely ill children.

Our finding that positive concordance never reached 100% even among wasted cases deviates from the MITS Alliance Guidelines.^10^ These results may be indicative of a need for a streamlined rollout of the guidelines or perhaps enhanced monitoring and evaluation of the implementation of the guidelines during the MITS examination. Our in-depth review of the CHAMPS data revealed instances where malnutrition was confirmed using photographs of the decedent. A future area of research might include a validation study for the use of photographs for determining or confirming malnutrition at death. However, to our knowledge, the use of photographs is not currently a validated method of assessing malnutrition, but perhaps with advancements in artificial intelligence, photographs may be a valuable tool for nutrition research.

This study enabled an inclusive examination of the proportion of deaths attributed to malnutrition via assessment of PAM or as determined by expert panel review. Variations in positive concordance existed by site, age and location of death, which may indicate an opportunity for future quality assurance of DeCoDe panels. Previous estimates of the contribution of malnutrition on U5M encompassed both anthropometric deficits, assessment of multiple micronutrients (including but not limited to vitamins A and B and zinc), and measures of dietary patterns. Until these data are collected in CHAMPS, current efforts to estimate the proportion of U5M attributable to malnutrition are likely underestimates of the true contribution of malnutrition to U5M. Future priority efforts in CHAMPS might include the investigation of tissue samples or serum for quantifying micronutrient malnutrition, indicators of maternal nutritional status, collection of gestational age and birth weight and data on dietary patterns.

## supplementary material

10.1136/bmjopen-2024-089874 online supplemental table 1

## Data Availability

Data are available upon reasonable request.
