# Supplementary material for: Importance of postmortem anthropometric evaluation in defining the role of malnutrition as a cause of infant and child deaths in Sub-Saharan Africa and South Asia: a cohort study
Source: BMJ Open. 2025 Feb 17;15(2):e089874. doi: 10.1136/bmjopen-2024-089874 (PMC11836817; doi:10.1136/bmjopen-2024-089874)
Supplement: online supplemental table 1 [file bmjopen-15-2-s001.pdf]

Supplemental Table

Supplemental Table 1. Causal chain classifications and ICD-10 codes for infant and child deaths with undernutrition or HIV-related wasting syndrome in the causal chain by age group, December 2016 to September 2023

	**Overall** N = 1,405	**1-5 months** N= 357	**6-11 months** N= 269	**12-23 months** N= 310	**24-59 months** N= 302
**Malnutrition or HIV-related wasting syndrome classified as:**					
Immediate cause of death	2.0 (0.1%)	0.0 (0.0%)	1.0 (0.3%)	0.0 (0.0%)	1.0 (0.3%)
Underlying cause of death	345.0 (24.6%)	60.0 (13.7%)	103.0 (35.5%)	126.0 (37.0%)	56.0 (16.7%)
Morbid cause of death	103.0 (7.3%)	30.0 (6.8%)	18.0 (6.2%)	28.0 (8.2%)	27.0 (8.0%)
Other significant causes	127.0 (9.0%)	38.0 (8.7%)	29.0 (10.0%)	28.0 (8.2%)	32.0 (9.5%)
**ICD-10 codes[1]:**					
E40: Kwashiorkor	50.0 (3.6%)	8.0 (1.8%)	13.0 (4.5%)	17.0 (5.0%)	12.0 (3.6%)
E41: Nutritional marasmus	200.0 (14.2%)	53.0 (12.1%)	51.0 (17.6%)	67.0 (19.6%)	29.0 (8.6%)
E42: Marasmic kwashiorkor	77.0 (5.5%)	10.0 (2.3%)	19.0 (6.6%)	28.0 (8.2%)	20.0 (6.0%)
E43: Unspecified severe protein-calorie malnutrition	59.0 (4.2%)	18.0 (4.1%)	18.0 (6.2%)	14.0 (4.1%)	9.0 (2.7%)
E44: Protein-calorie malnutrition of moderate and mild degree	37.0 (2.6%)	3.0 (0.7%)	13.0 (4.5%)	9.0 (2.6%)	12.0 (3.6%)
E44.0: Moderate protein-energy malnutrition	52.0 (3.7%)	8.0 (1.8%)	18.0 (6.2%)	14.0 (4.1%)	12.0 (3.6%)
E44.1: Mild protein-calorie malnutrition	1.0 (0.1%)	0.0 (0.0%)	0.0 (0.0%)	0.0 (0.0%)	1.0 (0.3%)
E45: Retarded development following protein-calorie malnutrition	13.0 (0.9%)	4.0 (0.9%)	3.0 (1.0%)	2.0 (0.6%)	4.0 (1.2%)
E46: Unspecified protein-calorie malnutrition	32.0 (2.3%)	17.0 (3.9%)	5.0 (1.7%)	5.0 (1.5%)	5.0 (1.5%)
B22.2: HIV disease resulting in wasting syndrome	58.0 (4.1%)	7.0 (1.6%)	11.0 (3.8%)	26.0 (7.6%)	14.0 (4.2%)

[1] There were 572 deaths with malnutrition as causal or significant condition. Deaths may have multiple conditions in the causal pathway (e.g., underlying, immediate, and antecedent), therefore the number of causes of death could exceed the number of deaths (and the sum would be >100%)
