# Supplementary material for: Importance of postmortem anthropometric evaluation in defining the role of malnutrition as a cause of infant and child deaths in Sub-Saharan Africa and South Asia: a cohort study
Source: BMJ Open. 2025 Feb 17;15(2):e089874. doi: 10.1136/bmjopen-2024-089874 (PMC11836817; doi:10.1136/bmjopen-2024-089874)
Supplement: Supplementary file 2 [file bmjopen-2024-089874.reviewer_comments.pdf]

PEER REVIEW HISTORY

BMJ Open publishes all reviews undertaken for accepted manuscripts. Reviewers are asked to complete a checklist review form and are provided with free text boxes to elaborate on their assessment. These free text comments are reproduced below.

ARTICLE DETAILS

Title (Provisional)

The Importance of Postmortem Anthropometric Evaluation in Defining the Role of Malnutrition as a Cause of Infant and Child Deaths in Sub-Saharan Africa and South Asia: A Cohort Study

Authors

Das, Priya Mehta-Gupta; Madewell, Zachary J.; Blau, Dianna; Whitney, Cynthia G.; Ramakrishnan, U; Stein, Aryeh D.; Young, Melissa; Suchdev, Parminder; Network, CHAMPS

VERSION 1 - REVIEW

**Reviewer**	**1**
**Name**	**Wutor , Baleng Mahama**
**Affiliation**	**Medical Research Council Unit The Gambia at the London School of Hygiene and Tropical Medicine**
**Date**	**26-Oct-2024**
**COI**	**None to declare**

The study is important as it seeks to contribute to a more accurate attribution of malnutrition as a cause of death of children under-5 in sub-Saharan Africa and South Asia. The study analyzed and compared causes of death determined by post-mortem anthropometry and those determined by a panel of experts.

Comments for the authors.

Abstract

1. Line 40, typo, “expert” not exert

Introduction

1. Page 9, line 8, typo, “than” not “then”

Materials and methods

1. Page 9, line45, Does CHAMPS use the WHO Verbal autopsy questionnaire? Or it has its own verbal autopsy questionnaire. If the latter is the case, it may be helpful to state if CHAMP’s instrument for conducting verbal autopsies has been validated.

Results

1. Page 15, line 22 and Table 1. 85.5% of the deaths were said to be preventable. It is not clear to me how the classification of a cause of death being preventable or not was made. It will be useful (probably under materials and methods) to describe how the DeCoDe panel determined which deaths were preventable.
2. Page 16, line 33/34, typo, seems to be missing "than", kindly check
3. Page 18, line 13, typo, "associated" seems to be repeated.

Discussion

1. Page 19, line 12, Not sure what the "[10]" reference to. If it is a citation, this should conform with the style used in the rest of the paper.

**Reviewer**	**2**
**Name**	**Kelson, Zoe**
**Affiliation**	**University of Exeter, Mathematics**
**Date**	**09-Nov-2024**
**COI**	**None**

This study assessed the alignment between postmortem anthropometric malnutrition (PAM) and expert panel attribution, including variations by PAM type and severity.

Reviewer comments:

"Data from 1,405 deaths in six African countries (aged 1-59 months, during 2016- 2023) were analyzed" [Abstract]

and

"Our final analytic sample size was N=1,405 infant and child deaths."

Can the authors please further comment on whether the included cohort can be considered to be representative?

"Based on our knowledge of the severity of illness and malnutrition in the study settings 24, we expanded our definition of the lower bound of biologically plausible values to include cases with z-scores -10 standard deviations (SD). "

Can the authors please confirm how many additional cases were classified in the 'severe' grouping as a result of expanding the definition?

"performed sensitivity analyses to examine differences in sample demographics, modeling patterns, and model fit statistics between analytic samples including and excluding possible PTB, LBW, or SGA cases...

Sensitivity analyses did not reveal any significant differences, therefore we proceeded with the more inclusive sample of 1,405 to ensure sufficient power for statistical modeling. "

These sensitivity analyses help to demonstrate the robustness of, and provide confidence in, the study findings.

"Data on birthweight (66.5% missing) and gestational age (99.0% missing), needed to classify cases as LBW, PTB, or SGA, were missing for most of our analytic sample"

Can the authors please comment on whether they consider missing data to be missing at random?

Can the authors please clarify how this missing data was handled in the analysis, and the above-mentioned sensitivity analysis?

"Pearson chi-square tests and univariable models were used to examine relationships between covariates, exposure, and outcomes of interest. "

Can the type of univariable model please be specified here?

"We used 2X2 Contingency tables to evaluate the relationship between presence or absences of PAM and DeCoDe panel attributed malnutrition as causal or another significant condition (Yes/No)...

More specifically, we examined sensitivity (Se), specificity (Sp), and positive predictive values (PPV, which we also refer to as positive concordance) between the two methods."

Can the authors please also consider reporting NPV and accuracy outcomes?

"Covariates evaluated as potential confounders included: age (in months), site, and sex (male or female)."

and

"We used a p-value < 0.05 when exploring potential confounders and their associations with both exposure and outcome. Confounders were also considered if sufficient evidence in existing literature warranted inclusion in the model."

Can site please be included as a hierarchical variable (i.e. in a multilevel model, with fixed or random effects) or applied using robust standard errors, to suitably adjust standard errors for the clustering nature of site in the data?

Is data on ethnicity or race available to be included in the study?

Can the authors please consider including comorbidities as potential confounders?

Did the authors consider exploring maternal factors, such as medical, lifestyle, and socioeconomic variables?

"Adjusted ordinal logistic regression estimated the odds of exert panel attribution of malnutrition as a causal or significant condition to death by type of PAM. " [Abstract]

and

"Ordinal logistic regression was used to quantify the relationship between type (underweight, wasting based on ZAC, wasting based on ZWFL, stunting) and 3-level severity of PAM (at-risk, moderate, and severe) and malnutrition reported as a causal or other significant condition to the death of the child as noted by DeCoDe panels."

Appropriate modelling methods have been applied.

"Between December 2016 and December 2023, 1,405 infant and child deaths between 1-59 months of age underwent both MITS and DeCoDe, with complete data on sex, postmortem length, and weight"

Can missing data, detailed in the Table and Figure footnotes, be assumed to be missing at random?

Can the authors please clarify how missing data was handled in each of the analyses?

"Nearly a quarter of cases were from Sierra Leone (24.6%), with the remaining 23.8%, 22.0%, 17.9%, 6.5%, and 5.3% from Kenya, South Africa, Mozambique, Mali, and Ethiopia, respectively. "

As well as adjusting for site in the modelling, can the authors please consider if a stratified analyses by country may add to the interpretation of the study findings?

"Table 1. Demographics, classification of postmortem anthropometric measurements, and Attrition of deaths to malnutrition for infant and child deaths enrolled in Child Health and Mortality Prevention Surveillance (CHAMPS), December 2016 to September 2023."

Can this data please also be shown by outcome categorisation?

"Severity of malnutrition based on ZWEI, ZAC, ZWFL, and ZLEN were all independently associated with malnutrition as a causal or significant condition in the death (Figure 1)."

To clarify, Figure 1 shows separate models for each malnutritution metric?

Can the authors please conduct a multivariate model that includes all metrics simultaneously? This will show which metrics are independently associated with malnutrition as a causal or significant condition in the death.

"The composite indicator of any moderate to severe postmortem anthropometric malnutrition was highly sensitive (97.2%, 95%CI: (95.5, 98.4)) but not specific (41.3%, 95% CI: 37.9, 44.7)"

and

"Figure 1 illustrates the association between type (underweight, wasting based on ZAC, wasting based on ZWFL, and stunting) and 3-level severity (at-risk, moderate, severe) of various PAM indicators and the odds of malnutrition being reported as a causal or significant condition in the death. In general, increasing severity of postmortem anthropometric malnutrition was associated with greater odds of DeCoDe panel attributed malnutrition as a causal or significant condition to death. "

Can the authors consider building a model, predicting DeCoDe outcome using all postmortem anthropometric malnutrition metrics as explanatory variables? This model could also include other potential predictors, such as sex and age? ROC curve analysis can be applied to assess predictive performance of this model?

"Our study is also subject to limitations. First, because measurements are taken only at the time of death, the measurement data alone does not allow inference of causality. Second, we cannot control the variability of antemortem data, which would have been helpful for determining more clearly if malnutrition had been present before the fatal illness. We also lacked data on gestational age and birthweight for many cases, critical for distinguishing between low birth weight, preterm birth, small for gestational age, intrauterine growth restriction. It is possible we are measuring relative validity of PAM against DeCoDe death attribution as the DeCoDe panels considers postmortem anthropometric measures in cause of death attribution. CHAMPS teams have a limited window between death and burial to enroll and sample deaths, leading to potential challenges in enrolling deaths outside catchment facilities 11,20,35; whether deaths not enrolled in CHAMPS are more or less likely to die from malnutrition than those who are enrolled needs further evaluation. Lastly, it is important to acknowledge the limitations of interpreting the postmortem anthropometry in a cohort of severely ill children."

The authors discuss the study limitations.

Did the authors adhere to any guidelines for the reporting of this study? If so, can the associated checklist please be provided?

VERSION 1 - AUTHOR RESPONSE

<u>**Reviewer 1**</u>:
Dr. Baleng Mahama Wutor, Medical Research Council Unit The Gambia at the London School of Hygiene and Tropical Medicine

<u>**Comments to the Author:**</u>
The paper is well written, and the data presented appropriately. Find below my comments.

<u>**Abstract**</u>
Line 40, typo, "expert" not exert
Thank you for catching this typo. We have corrected this error. Please see line 85 in Main Document – Marked Copy.

<u>**Introduction**</u>
Page 9, line 8, typo, "than" not "then"
Thank you for catching this typo. We have corrected this error. Please see line 167 in Main Document – Marked Copy.

<u>**Materials and methods**</u>

Page 9, line 45, Does CHAMPS use the WHO Verbal autopsy questionnaire? Or it has its own verbal autopsy questionnaire. If the latter is the case, it may be helpful to state if CHAMP's instrument for conducting verbal autopsies has been validated.
Yes, a citation has been provided on page line 187 of the methods section. We have cited the following publication as well.

Assefa N, Scott A, Madrid L, et al. Comparison of causes of stillbirth and child deaths as determined by verbal autopsy and minimally invasive tissue sampling. PLOS Global Public Health. 2024;4(7):e0003065. doi:10.1371/journal.pgph.0003065
https://journals.plos.org/globalpublichealth/article?id=10.1371/journal.pgph.0003065

Results
Page 15, line 22 and Table 1. 85.5% of the deaths were said to be preventable. It is not clear to me how the classification of a cause of death being preventable or not was made. It will be useful (probably under materials and methods) to describe how the DeCoDe panel determined which deaths were preventable.

We thank the reviewer for this feedback. We have linked out to a publication to provide these details. We have provided the following citation that provides additional detail.

Please see Madewell ZJ, Whitney CG, Velaphi S, et al. Prioritizing Health Care Strategies to Reduce Childhood Mortality. JAMA Network Open. 2022;5(10):e2237689. doi:10.1001/jamanetworkopen.2022.37689
https://jamanetwork.com/journals/jamanetworkopen/fullarticle/2797547

We also added text in lines 185–189:
"For each death, the DECODE panel determined whether the death was preventable (yes, no, or under certain circumstances) by considering all the information available for each child, which may include demographic, clinical, pathological, microbiological, WHO's verbal autopsy [21], photography, and anthropometric measurements. If the panel deems a death to be preventable, they are referring to the conditions immediately surrounding the death of that child" and not the broader social context. The details of this process have been published elsewhere [22].

Page 16, line 33/34, typo, seems to be missing "than", kindly check.
Thank you for catching this typo. We have corrected this and added "also had". Please see line 339 in Main Document – Marked Copy.

Page 18, line 13, typo, "associated" seems to be repeated.
Thank you for catching this typo. We have corrected this. Please see line 372 in Main Document – Marked Copy.

Discussion
Page 19, line 12, Not sure what the "[10]" reference to. If it is a citation, this should conform with the style used in the rest of the paper.
Thank you for catching this typo. We have corrected this error and inserted the correct

citation in the appropriate format. Please see lines 144, 391, and 468 in Main Document – Marked Copy.

Reviewer 2:

Prof. Zoe Kelson, University of Exeter

Comments to the Author:

This study assessed the alignment between postmortem anthropometric malnutrition (PAM) and expert panel attribution, including variations by PAM type and severity.

Reviewer comments:

"Data from 1,405 deaths in six African countries (aged 1-59 months, during 2016- 2023) were analyzed" [Abstract] and "Our final analytic sample size was N=1,405 infant and child deaths."

Can the authors please further comment on whether the included cohort can be considered to be representative?

We thank the reviewer for this feedback. These data come from a surveillance system that is intended to capture all deaths. Limitations of the CHAMPS methodology has been documented in great detail in the publications below:

- Salzberg NT, Sivalogan K, Bassat Q, et al; Child Health and Mortality Prevention Surveillance (CHAMPS) Methods Consortium. Mortality surveillance methods to identify and characterize deaths in child health and mortality prevention surveillance network sites. *Clin Infect Dis*. 2019;69(suppl 4):S262-S273.

- Blau DM, Caneer JP, Philipsborn RP, et al. Overview and development of the child health and mortality prevention surveillance determination of cause of death (decode) process and decode diagnosis standards. *Clin Infect Dis*. 2019;69(suppl 4):S333-S341.

- Breiman RF, Blau DM, Mutevedzi P, et al; CHAMPS Consortium. Postmortem investigations and identification of multiple causes of child deaths: an analysis of findings from the Child Health and Mortality Prevention Surveillance (CHAMPS) network. *PLoS Med*. 2021;18(9):e1003814.

One such limitation is the generalizability of these data. Currently, these data are not considered to be representative. To address this limitation, we have added the following sentence to Discussion, starting on line 445–451:

"While the inclusion of data from six high-mortality countries enhances generalizability to similar settings in Sub-Saharan Africa, the cohort may not fully represent all under-5 deaths within these regions or globally. Facility-based deaths are overrepresented due to CHAMPS' reliance on timely death identification for MITS, potentially biasing findings toward families with better healthcare access. Additionally, variability in site-specific epidemiology and malnutrition attribution by DeCoDe panels may limit the comparability of findings across sites"

We have also provided the following citation [37]:

Bassat Q, Blau DM, Ogbuanu IU, et al. Causes of Death Among Infants and Children in the Child Health and Mortality Prevention Surveillance (CHAMPS) Network. *JAMA Netw Open.* 2023;6(7):e2322494. doi:10.1001/jamanetworkopen.2023.22494

"Based on our knowledge of the severity of illness and malnutrition in the study settings 24, we expanded our definition of the lower bound of biologically plausible values to include cases with z-scores -10 standard deviations (SD). "

Can the authors please confirm how many additional cases were classified in the 'severe' grouping as a result of expanding the definition?

We thank the reviewer for this feedback. This number varies by which anthropometric indicator we examined. This is a result of variations what was deemed to be biologically implausible by anthropometric index and variations in the age of the child included in the original study (For example, biologically implausible values for ZLEN and ZWEI = -6 SD vs. ZWFL and ZAC = -5 SD, additionally in the original study ZAC was assessed only among children 3-59 months of age.

**Z score indicator**	**Extended definition of BIV**	**Additional cases included**
ZLEN	-6 to -10	99
ZWEI	-6 to -10	133
ZWFL	-5 to -10	203
ZAC	-5 to -10	132

"performed sensitivity analyses to examine differences in sample demographics, modeling patterns, and model fit statistics between analytic samples including and excluding possible PTB, LBW, or SGA cases...

Sensitivity analyses did not reveal any significant differences, therefore we proceeded with the more inclusive sample of 1,405 to ensure sufficient power for statistical modeling. "

These sensitivity analyses help to demonstrate the robustness of, and provide confidence in, the study findings.

Thank you for noting this!

"Data on birthweight (66.5% missing) and gestational age (99.0% missing), needed to classify cases as LBW, PTB, or SGA, were missing for most of our analytic sample".

Can the authors please comment on whether they consider missing data to be missing at random? Can the authors please clarify how this missing data was handled in the analysis, and the above-mentioned sensitivity analysis?

We thank the reviewer for their feedback. We ran multiple sensitivity analyses examining sociodemographic characteristics of analytic samples with and without complete data on sex, length/height, or weight. We also included and excluded cases with data to confirm low birthweight, preterm birthweight, or small- for-gestational age. We did not find significant differences between these samples and therefore opted to include the most inclusive sample for our analyses. Given these sensitivity analyses we assume missing data to be missing at random or a limitation of the study design.

"Pearson chi-square tests and univariable models were used to examine relationships between covariates, exposure, and outcomes of interest. "

Can the type of univariable model please be specified here?

We have corrected the text “Pearson chi-square tests were used to examine relationships between covariates, exposure, and outcomes of interest”. Please see line 265–266 in Main Document – Marked Copy. Details of our modeling approach can be referenced starting on line 287 in Main Document – Marked Copy.

"We used 2X2 Contingency tables to evaluate the relationship between presence or absences of PAM and DeCoDe panel attributed malnutrition as causal or another significant condition (Yes/No)...More specifically, we examined sensitivity (Se), specificity (Sp), and positive predictive values (PPV, which we also refer to as positive concordance) between the two methods."

Can the authors please also consider reporting NPV and accuracy outcomes?

We thank the reviewer for this feedback. Initially we examined Se, Sp, PPV, NPV, as well as diagnostic accuracy. These results are presented for the reviewer below.

Upon review of these findings, our study team decided to only present sensitivity, specificity, and positive predictive values as they are the most relevant from a clinical standpoint.

Table 2. Performance of Various Postmortem Anthropometric Indices to Determine Malnutrition as a Causal or Other Significant Condition among Infant and Child in the Child Health and Mortality Prevention Surveillance Network (CHAMPS) December 2016 to September 2023

		DeCoDe[1] Attributed Malnutrition						
		Yes	No	SE[3]	SP[3]	PPV[3]	NPV[3]	DA[3]
Underweight[2]	Yes	513	324	89.70%	60.60%	61.30%	89.40%	72.50%
	No	59	499					
		DeCoDe Attributed Malnutrition						
		Yes	No	SE	SP	PPV	NPV	DA
Wasted[4]	Yes	391	118	77.00%	81.90%	76.80%	82.00%	79.70%
	No	117	533					
		DeCoDe Attributed Malnutrition						
		Yes	No	SE	SP	PPV	NPV	DA
Wasted[5]	Yes	453	268	82.70%	64.90%	62.80%	83.90%	72.30%
	No	95	495					
		DeCoDe Attributed Malnutrition						
		Yes	No	SE	SP	PPV	NPV	DA
Stunted[6]	Yes	340	240	60.50%	70.30%	58.60%	71.90%	66.30%
	No	222	567					
		DeCoDe Attributed Malnutrition						
		Yes	No	SE	SP	PPV	NPV	DA
Any Moderate to Severe Malnutrition[7]	Yes	556	489	97.20%	41.30%	53.20%	95.60%	64.10%
	No	16	344					

"Covariates evaluated as potential confounders included: age (in months), site, and sex (male or female)." And "We used a p-value < 0.05 when exploring potential confounders and their associations with both exposure and outcome. Confounders were also considered if sufficient evidence in existing literature warranted inclusion in the model."

Can site please be included as a hierarchical variable (i.e. in a multilevel model, with fixed or random effects) or applied using robust standard errors, to suitably adjust standard errors for the clustering nature of site in the data?

We thank the reviewer for this feedback. A hierarchical model was considered however, we felt that because sites were predetermined, they are more likely to be included in the model

as strata and to maintain a parsimonious model, we chose not to move forward with site as a hierarchical variable.

Is data on ethnicity or race available to be included in the study?
No, unfortunately data on race and ethnicity are not available.

Can the authors please consider including comorbidities as potential confounders?
We thank the reviewer for this suggestion. These data are not readily available in the clinical abstraction datafiles.

Did the authors consider exploring maternal factors, such as medical, lifestyle, and socioeconomic variables?
Yes, we considered maternal, medical, environmental, and economic variables in detail prior to starting the analysis (DAGs). However, due to limited data availability inclusion of these data were not possible in this analysis. Future longitudinal data collection may allow us to better explore maternal factors and socioeconomic factors. Prospective pregnancy surveillance is currently ongoing in the CHAMPS sites, we hope to incorporate these data in the future.

"Adjusted ordinal logistic regression estimated the odds of exert panel attribution of malnutrition as a causal or significant condition to death by type of PAM. " [Abstract] and "Ordinal logistic regression was used to quantify the relationship between type (underweight, wasting based on ZAC, wasting based on ZWFL, stunting) and 3-level severity of PAM (at-risk, moderate, and severe) and malnutrition reported as a causal or other significant condition to the death of the child as noted by DeCoDe panels."
Appropriate modelling methods have been applied.
Thank you for noting this!

"Between December 2016 and December 2023, 1,405 infant and child deaths between 1-59 months of age underwent both MITS and DeCoDe, with complete data on sex, postmortem length, and weight"
Can missing data, detailed in the Table and Figure footnotes, be assumed to be missing at random?
There were 20 cases missing data on sex, postmortem length or weight, thereby increasing our analytic sample from 1,405 to 1,425. There were no significant differences in demographics or prevalence of malnutrition or severity of malnutrition between the two samples. For these reasons we assume the data are missing at random.

Can the authors please clarify how missing data was handled in each of the analyses? MAR, detail how data missingness is a limitation of our study.
We thank the reviewer for their feedback. We ran multiple sensitivity analyses examining sociodemographic characteristics of analytic samples with and without complete data on sex, length/height, or weight. We also included and excluded cases with data to confirm low birthweight, preterm birthweight, or small- for-gestational age. We did not find significant differences between these samples and therefore opted to include the most inclusive sample for our analyses. Given these sensitivity analyses we assume missing data to be missing at random. We have added additional text in the data missingness section to

address this concern starting on line 258–261 in Main Document – Marked Copy.

"Nearly a quarter of cases were from Sierra Leone (24.6%), with the remaining 23.8%, 22.0%, 17.9%, 6.5%, and 5.3% from Kenya, South Africa, Mozambique, Mali, and Ethiopia, respectively. "

As well as adjusting for site in the modelling, can the authors please consider if a stratified analyses by country may add to the interpretation of the study findings?

We thank the reviewer for this feedback. At this time, we believe that stratified analyses will not be possible given sample size limitations by site. We plan to conduct stratified analyses in the future.

"Table 1. Demographics, classification of postmortem anthropometric measurements, and Attrition of deaths to malnutrition for infant and child deaths enrolled in Child Health and Mortality Prevention Surveillance (CHAMPS), December 2016 to September 2023."

Can this data please also be shown by outcome categorization?

We thank the reviewer for this suggestion. At this time, we do not feel this would enhance this analysis. We feel adjustment for site is sufficient.

"Severity of malnutrition based on ZWEI, ZAC, ZWFL, and ZLEN were all independently associated with malnutrition as a causal or significant condition in the death (Figure 1)."

To clarify, Figure 1 shows separate models for each malnutrition metric?

Can the authors please conduct a multivariate model that includes all metrics simultaneously? This will show which metrics are independently associated with malnutrition as a causal or significant condition in the death.

We thank the reviewer for this feedback. These models were included in our exploratory analyses. We examined these relationships in multiple models, including those inclusive of all metrics and discussed which models were to be included in the final manuscript. Inclusion of all metrics did not significantly improve model statistics and patterns were comparable.

"The composite indicator of any moderate to severe postmortem anthropometric malnutrition was highly sensitive (97.2%, 95%CI: (95.5, 98.4)) but not specific (41.3%, 95% CI: 37.9, 44.7)"

and "Figure 1 illustrates the association between type (underweight, wasting based on ZAC, wasting based on ZWFL, and stunting) and 3-level severity (at-risk, moderate, severe) of various PAM indicators and the odds of malnutrition being reported as a causal or significant condition in the death. In general, increasing severity of postmortem anthropometric malnutrition was associated with greater odds of DeCoDe panel attributed malnutrition as a causal or significant condition to death.”

Can the authors consider building a model, predicting DeCoDe outcome using all postmortem anthropometric malnutrition metrics as explanatory variables? This model could also include other potential predictors, such as sex and age? ROC curve analysis can be applied to assess predictive performance of this model?

We thank the reviewer for this feedback. These models were included in our exploratory analyses. Results were like the models we chose to present in the paper and model statistics did not show significant improvement.

"Our study is also subject to limitations. First, because measurements are taken only at the time of death, the measurement data alone does not allow inference of causality. Second, we cannot control the variability of antemortem data, which would have been helpful for determining more clearly if malnutrition had been present before the fatal illness. We also lacked data on gestational age and birthweight for many cases, critical for distinguishing between low birth weight, preterm birth, small for gestational age, intrauterine growth restriction. It is possible we are measuring relative validity of PAM against DeCoDe death attribution as the DeCoDe panels considers postmortem anthropometric measures in cause of death attribution. CHAMPS teams have a limited window between death and burial to enroll and sample deaths, leading to potential challenges in enrolling deaths outside catchment facilities 11,20,35; whether deaths not enrolled in CHAMPS are more or less likely to die from malnutrition than those who are enrolled needs further evaluation. Lastly, it is important to acknowledge the limitations of interpreting the postmortem anthropometry in a cohort of severely ill children."
The authors discuss the study limitations.
Thank you for noting this!

VERSION 2 - AUTHOR RESPONSE

Reviewer 1:
Dr. Baleng Mahama Wutor, Medical Research Council Unit The Gambia at the London School of Hygiene and Tropical Medicine

Comments to the Author:
The paper is well written, and the data presented appropriately. Find below my comments.

Abstract
Line 40, typo, "expert" not exert
Thank you for catching this typo. We have corrected this error. Please see line 85 in Main Document – Marked Copy.

Introduction
Page 9, line 8, typo, "than" not "then"
Thank you for catching this typo. We have corrected this error. Please see line 167 in Main Document – Marked Copy.

Materials and methods
Page 9, line 45, Does CHAMPS use the WHO Verbal autopsy questionnaire? Or it has its own verbal autopsy questionnaire. If the latter is the case, it may be helpful to state if CHAMP's instrument for conducting verbal autopsies has been validated.
Yes, a citation has been provided on page line 187 of the methods section. We have cited the following publication as well.

Assefa N, Scott A, Madrid L, et al. Comparison of causes of stillbirth and child deaths as determined by verbal autopsy and minimally invasive tissue sampling. PLOS Global Public Health. 2024;4(7):e0003065. doi:10.1371/journal.pgph.0003065 https://journals.plos.org/globalpublichealth/article?id=10.1371/journal.pgph.0003065

Results

Page 15, line 22 and Table 1. 85.5% of the deaths were said to be preventable. It is not clear to me how the classification of a cause of death being preventable or not was made. It will be useful (probably under materials and methods) to describe how the DeCoDe panel determined which deaths were preventable.

We thank the reviewer for this feedback. We have linked out to a publication to provide these details. We have provided the following citation that provides additional detail.

Please see Madewell ZJ, Whitney CG, Velaphi S, et al. Prioritizing Health Care Strategies to Reduce Childhood Mortality. JAMA Network Open. 2022;5(10):e2237689. doi:10.1001/jamanetworkopen.2022.37689 https://jamanetwork.com/journals/jamanetworkopen/fullarticle/2797547

We also added text in lines 185–189:
"For each death, the DECODE panel determined whether the death was preventable (yes, no, or under certain circumstances) by considering all the information available for each child, which may include demographic, clinical, pathological, microbiological, WHO's verbal autopsy [21], photography, and anthropometric measurements. If the panel deems a death to be preventable, they are referring to the conditions immediately surrounding the death of that child" and not the broader social context. The details of this process have been published elsewhere [22].

Page 16, line 33/34, typo, seems to be missing "than", kindly check.
Thank you for catching this typo. We have corrected this and added "also had". Please see line 339 in Main Document – Marked Copy.

Page 18, line 13, typo, "associated" seems to be repeated.
Thank you for catching this typo. We have corrected this. Please see line 372 in Main Document – Marked Copy.

Discussion

Page 19, line 12, Not sure what the "[10]" reference to. If it is a citation, this should conform with the style used in the rest of the paper.
Thank you for catching this typo. We have corrected this error and inserted the correct citation in the appropriate format. Please see lines 144, 391, and 468 in Main Document – Marked Copy.

Reviewer 2:

Prof. Zoe Kelson, University of Exeter

Comments to the Author:
This study assessed the alignment between postmortem anthropometric malnutrition (PAM) and expert panel attribution, including variations by PAM type and severity.

Reviewer comments:

"Data from 1,405 deaths in six African countries (aged 1-59 months, during 2016- 2023) were analyzed" [Abstract] and "Our final analytic sample size was N=1,405 infant and child deaths." **Can the authors please further comment on whether the included cohort can be considered to be representative?**
We thank the reviewer for this feedback. These data come from a surveillance system that is intended to capture all deaths. Limitations of the CHAMPS methodology has been documented in great detail in the publications below:

- Salzberg NT, Sivalogan K, Bassat Q, et al; Child Health and Mortality Prevention Surveillance (CHAMPS) Methods Consortium. Mortality surveillance methods to identify and characterize deaths in child health and mortality prevention surveillance network sites. *Clin Infect Dis*. 2019;69(suppl 4):S262-S273.

- Blau DM, Caneer JP, Philipsborn RP, et al. Overview and development of the child health and mortality prevention surveillance determination of cause of death (decode) process and decode diagnosis standards. *Clin Infect Dis*. 2019;69(suppl 4):S333-S341.

- Breiman RF, Blau DM, Mutevedzi P, et al; CHAMPS Consortium. Postmortem investigations and identification of multiple causes of child deaths: an analysis of findings from the Child Health and Mortality Prevention Surveillance (CHAMPS) network. *PLoS Med*. 2021;18(9):e1003814.

One such limitation is the generalizability of these data. Currently, these data are not considered to be representative. To address this limitation, we have added the following sentence to Discussion, starting on line 445–451:

“While the inclusion of data from six high-mortality countries enhances generalizability to similar settings in Sub-Saharan Africa, the cohort may not fully represent all under-5 deaths within these regions or globally. Facility-based deaths are overrepresented due to CHAMPS’ reliance on timely death identification for MITS, potentially biasing findings toward families with better healthcare access. Additionally, variability in site-specific epidemiology and malnutrition attribution by DeCoDe panels may limit the comparability of findings across sites”

We have also provided the following citation [37]:
Bassat Q, Blau DM, Ogbuanu IU, et al. Causes of Death Among Infants and Children in the Child

Health and Mortality Prevention Surveillance (CHAMPS) Network. *JAMA Netw Open.* 2023;6(7):e2322494. doi:10.1001/jamanetworkopen.2023.22494

"Based on our knowledge of the severity of illness and malnutrition in the study settings 24, we expanded our definition of the lower bound of biologically plausible values to include cases with z-scores -10 standard deviations (SD). "

Can the authors please confirm how many additional cases were classified in the 'severe' grouping as a result of expanding the definition?

We thank the reviewer for this feedback. This number varies by which anthropometric indicator we examined. This is a result of variations what was deemed to be biologically implausible by anthropometric index and variations in the age of the child included in the original study (For example, biologically implausible values for ZLEN and ZWEI = -6 SD vs. ZWFL and ZAC = -5 SD, additionally in the original study ZAC was assessed only among children 3-59 months of age.

Z score indicator	Extended definition of BIV	Additional cases included
ZLEN	-6 to -10	99
ZWEI	-6 to -10	133
ZWFL	-5 to -10	203
ZAC	-5 to -10	132

"performed sensitivity analyses to examine differences in sample demographics, modeling patterns, and model fit statistics between analytic samples including and excluding possible PTB, LBW, or SGA cases...

Sensitivity analyses did not reveal any significant differences, therefore we proceeded with the more inclusive sample of 1,405 to ensure sufficient power for statistical modeling. "

These sensitivity analyses help to demonstrate the robustness of, and provide confidence in, the study findings. Thank you for noting this!

"Data on birthweight (66.5% missing) and gestational age (99.0% missing), needed to classify cases as LBW, PTB, or SGA, were missing for most of our analytic sample".

Can the authors please comment on whether they consider missing data to be missing at random? Can the authors please clarify how this missing data was handled in the analysis, and the above-mentioned sensitivity analysis?

We thank the reviewer for their feedback. We ran multiple sensitivity analyses examining sociodemographic characteristics of analytic samples with and without complete data on sex, length/height, or weight. We also included and excluded cases with data to confirm low birthweight, preterm birthweight, or small- for-gestational age. We did not find significant differences between these samples and therefore opted to include the most inclusive sample for our analyses. Given these sensitivity analyses we assume missing data to be missing at random or a limitation of the study design.

"Pearson chi-square tests and univariable models were used to examine relationships between covariates, exposure, and outcomes of interest. "

Can the type of univariable model please be specified here?

We have corrected the text "Pearson chi-square tests were used to examine relationships between covariates, exposure, and outcomes of interest". Please see line 265–266 in Main Document – Marked Copy. Details of our modeling approach can be referenced starting on line 287 in Main Document – Marked Copy.

"We used 2X2 Contingency tables to evaluate the relationship between presence or absences of

PAM and DeCoDe panel attributed malnutrition as causal or another significant condition (Yes/No)...More specifically, we examined sensitivity (Se), specificity (Sp), and positive predictive values (PPV, which we also refer to as positive concordance) between the two methods."

Can the authors please also consider reporting NPV and accuracy outcomes?

We thank the reviewer for this feedback. Initially we examined Se, Sp, PPV, NPV, as well as diagnostic accuracy. These results are presented for the reviewer below.

Upon review of these findings, our study team decided to only present sensitivity, specificity, and positive predictive values as they are the most relevant from a clinical standpoint.

Table 2. Performance of Various Postmortem Anthropometric Indices to Determine Malnutrition as a Causal or Other Significant Condition among Infant and Child in the Child Health and Mortality Prevention Surveillance Network (CHAMPS) December 2016 to September 2023

		DeCoDe[2] Attributed Malnutrition						
		Yes	No	SE[3]	SP[3]	PPV[3]	NPV[3]	DA[3]
Underweight[2]	Yes	513	324	89.70%	60.60%	61.30%	89.40%	72.50%
	No	59	499					
		DeCoDe Attributed Malnutrition						
		Yes	No	SE	SP	PPV	NPV	DA
Wasted[4]	Yes	391	118	77.00%	81.90%	76.80%	82.00%	79.70%
	No	117	533					
		DeCoDe Attributed Malnutrition						
		Yes	No	SE	SP	PPV	NPV	DA
Wasted[5]	Yes	453	268	82.70%	64.90%	62.80%	83.90%	72.30%
	No	95	495					
		DeCoDe Attributed Malnutrition						
		Yes	No	SE	SP	PPV	NPV	DA
Stunted[6]	Yes	340	240	60.50%	70.30%	58.60%	71.90%	66.30%
	No	222	567					
		DeCoDe Attributed Malnutrition						
		Yes	No	SE	SP	PPV	NPV	DA
Any Moderate to Severe Malnutrition[7]	Yes	556	489	97.20%	41.30%	53.20%	95.60%	64.10%
	No	16	344					

"Covariates evaluated as potential confounders included: age (in months), site, and sex (male or female)." And "We used a p-value < 0.05 when exploring potential confounders and their associations with both exposure and outcome. Confounders were also considered if sufficient evidence in existing literature warranted inclusion in the model."

Can site please be included as a hierarchical variable (i.e. in a multilevel model, with fixed or random effects) or applied using robust standard errors, to suitably adjust standard errors for the clustering nature of site in the data?
We thank the reviewer for this feedback. A hierarchical model was considered however, we felt that because sites were predetermined, they are more likely to be included in the model as strata and to maintain a parsimonious model, we chose not to move forward with site as a hierarchical variable.

Is data on ethnicity or race available to be included in the study?
No, unfortunately data on race and ethnicity are not available.

Can the authors please consider including comorbidities as potential confounders? We thank the reviewer for this suggestion. These data are not readily available in the clinical abstraction datafiles.

Did the authors consider exploring maternal factors, such as medical, lifestyle, and socioeconomic variables?
Yes, we considered maternal, medical, environmental, and economic variables in detail prior to starting the analysis (DAGs). However, due to limited data availability inclusion of these data were not possible in this analysis. Future longitudinal data collection may allow us to better explore maternal factors and socioeconomic factors. Prospective pregnancy surveillance is currently ongoing in the CHAMPS sites, we hope to incorporate these data in the future.

"Adjusted ordinal logistic regression estimated the odds of exert panel attribution of malnutrition as a causal or significant condition to death by type of PAM. " [Abstract]
and "Ordinal logistic regression was used to quantify the relationship between type (underweight, wasting based on ZAC, wasting based on ZWFL, stunting) and 3-level severity of PAM (at-risk, moderate, and severe) and malnutrition reported as a causal or other significant
condition to the death of the child as noted by DeCoDe panels."
Appropriate modelling methods have been applied.
Thank you for noting this!

"Between December 2016 and December 2023, 1,405 infant and child deaths between 1-59 months of age underwent both MITS and DeCoDe, with complete data on sex, postmortem length, and weight"
Can missing data, detailed in the Table and Figure footnotes, be assumed to be missing at random?
There were 20 cases missing data on sex, postmortem length or weight, thereby increasing our analytic sample from 1,405 to 1,425. There were no significant differences in demographics or prevalence of malnutrition or severity of malnutrition between the two samples. For these reasons we assume the data are missing at random.

Can the authors please clarify how missing data was handled in each of the analyses?
MAR, detail how data missingness is a limitation of our study.

We thank the reviewer for their feedback. We ran multiple sensitivity analyses examining sociodemographic characteristics of analytic samples with and without complete data on sex, length/height, or weight. We also included and excluded cases with data to confirm low birthweight, preterm birthweight, or small- for-gestational age. We did not find significant differences between these samples and therefore opted to include the most inclusive sample for our analyses. Given these sensitivity analyses we assume missing data to be missing at random. We have added additional text in the data missingness section to address this concern starting on line 258–261 in Main Document – Marked Copy.

"Nearly a quarter of cases were from Sierra Leone (24.6%), with the remaining 23.8%, 22.0%, 17.9%, 6.5%, and 5.3% from Kenya, South Africa, Mozambique, Mali, and Ethiopia, respectively.
"
As well as adjusting for site in the modelling, can the authors please consider if a stratified analyses by country may add to the interpretation of the study findings?
We thank the reviewer for this feedback. To explore the relationship between PAM and the DeCoDe panel's attribution of malnutrition, potential interactions across key variables were assessed. However, interactions were inconsistent, with variability observed for site and age in the model examining stunting, for sex in the model assessing underweight, and for age in the model evaluating wasting. Additionally, when exposure was defined as any malnutrition, no significant interactions were detected. Due to this inconsistency, a stratified analysis was not conducted, as the lack of consistent interaction patterns limited the justification for stratification. We greatly value the feedback and will keep this in mind as our data expands and future analyses are conducted. Please see lines 292–295 in Main Document – Marked Copy.

"Table 1. Demographics, classification of postmortem anthropometric measurements, and Attrition of deaths to malnutrition for infant and child deaths enrolled in Child Health and Mortality Prevention Surveillance (CHAMPS), December 2016 to September 2023." **Can this data please also be shown by outcome categorization?**
We thank the reviewer for this suggestion. Please see response above. Please see lines 292–295 in Main Document – Marked Copy.

"Severity of malnutrition based on ZWEI, ZAC, ZWFL, and ZLEN were all independently associated with malnutrition as a causal or significant condition in the death (Figure 1)." To clarify, Figure 1 shows separate models for each malnutrition metric? **Can the authors please conduct a multivariate model that includes all metrics simultaneously? This will show which metrics are independently associated with malnutrition as a causal or significant condition in the death.**
We thank the reviewer for this feedback. These models were included in our exploratory analyses. We examined these relationships in multiple models, including those inclusive of all metrics and discussed which models were to be included in the final manuscript. We also examined a multivariate model which included all measures of PAM. The inclusion of all metrics did not alter the pattern described in detail above. Concerns regarding collinearity between PAM indices and differences in the ages of children captured in the multivariate model further justified the

author's decision to exclude the model. Inclusion of all metrics did not significantly improve model statistics and patterns were comparable. Please see additional details in the table below:

	Exposure	At risk	Moderate	Severe
Models included in the paper	Underweight (ZWEI)	2.2 (1.2, 3.9), p=0.008	10.5 (6.3, 18.1), p<0.001	42.5 (26.6, 70.9), p<0.001
	Severe acute malnutrition (ZAC; wasting)	4.1 (2.3, 6.3), p<0.001	16.2 (10.0, 26.7), p<0.001	49.7 (31.6, 80.3), p<0.001
	Wasting (ZWFL)	1.70 (1.1, 2.8), p=0.024	7.4 (4.7, 11.7), p<0.001	19.6 (13.1, 29.8), p<0.001
	Stunting (ZLEN)	2.5 (1.8, 3.5), p=<0.001	5.4 (3.7, 7.88), p<0.001	6.5 (4.6, 9.0), p<0.001
1.7 (0.8, 3.6), p=0.2			4.6 (2.1, 10.5), p<0.001	7.1 (2.8, 19.0), p<0.001
Models not shown	All PAM indices	2.1 (1.3, 3.5), p=0.005	5.8 (3.2, 10.5), p<0.001	10.7 (5.7, 20.5), p<0.001
0.9 (0.5, 1.8), p=0.8			2.6 (1.3, 5.2), p=0.007	2.1 (1.0, 4.7), p=0.057
1.1 (0.5, 2.1), p=0.9		0.97 (0.6, 1.6), p>0.9	2.1 (1.1, 3.98), p=0.017	

"The composite indicator of any moderate to severe postmortem anthropometric malnutrition was highly sensitive (97.2%, 95%CI: (95.5, 98.4)) but not specific (41.3%, 95% CI: 37.9, 44.7)" and "Figure 1 illustrates the association between type (underweight, wasting based on ZAC, wasting based on ZWFL, and stunting) and 3-level severity (at-risk, moderate, severe) of various PAM indicators and the odds of malnutrition being reported as a causal or

significant condition in the death. In general, increasing severity of postmortem anthropometric malnutrition was associated with greater odds of DeCoDe panel attributed malnutrition as a causal or significant condition to death.”

Can the authors consider building a model, predicting DeCoDe outcome using all postmortem anthropometric malnutrition metrics as explanatory variables? This model could also include other potential predictors, such as sex and age? ROC curve analysis can be applied to assess predictive performance of this model?

We thank the reviewer for this feedback. These models were included in our exploratory analyses. Results were like the models we chose to present in the paper and model statistics did not show significant improvement.

"Our study is also subject to limitations. First, because measurements are taken only at the time of death, the measurement data alone does not allow inference of causality. Second, we cannot control the variability of antemortem data, which would have been helpful for determining more clearly if malnutrition had been present before the fatal illness. We also lacked data on gestational age and birthweight for many cases, critical for distinguishing between low birth weight, preterm birth, small for gestational age, intrauterine growth restriction. It is possible we are measuring relative validity of PAM against DeCoDe death attribution as the DeCoDe panels considers postmortem anthropometric measures in cause of death attribution. CHAMPS teams have a limited window between death and burial to enroll and sample deaths, leading to potential challenges in enrolling deaths outside catchment facilities 11,20,35; whether deaths not enrolled in CHAMPS are more or less likely to die from malnutrition than those who are enrolled needs further evaluation. Lastly, it is important to acknowledge the limitations of interpreting the postmortem anthropometry in a cohort of severely ill children." **The authors discuss the study limitations.**

Thank you for noting this!
